# Increases in skin perfusion and blood oxygen in the non‐exercising human limbs during exercise in the heat: Implications for control of circulation

**DOI:** 10.1113/EP092742

**Published:** 2025-06-18

**Authors:** Steven J. Trangmar, José González‐Alonso

**Affiliations:** ^1^ Carnegie School of Sport Leeds Beckett University Leeds UK; ^2^ Department of Sport, Health and Exercise Sciences, College of Health, Medicine and Life Sciences Brunel University of London Uxbridge UK

**Keywords:** blood oxygen, exercise, forearm blood flow

## Abstract

Blood flow in the inactive limb tissues and skin is widely thought to decline during incremental exercise to exhaustion due to augmented sympathoadrenal vasoconstrictor activity, but direct evidence to support this view is lacking. Here, we investigated the inactive‐forearm haemodynamic (${\dot{Q}}_{\mathrm{forearm}}$) and oxygenation responses to a range of two‐leg exercise intensities and durations in the heat. Blood oxygen and flow were measured in the forearm tissue and skin of endurance‐trained males during three incremental cycling exercise tests, with tests 1 and 2 separated by a 2 h bout of moderate constant load cycling exercise, all performed in the heat (35°C, 50% relative humidity, with fan cooling). In incremental exercise tests 1 and 3, ${\dot{Q}}_{\mathrm{forearm}}$ was stable from rest to ∼40% *W*_peak_, before increasing by ∼118% at 80% *W*_peak_ (*P* <0.001). Correspondingly, forearm skin arterio‐venous oxygen difference (a‐vO_2_ diff) decreased by ∼62% at 80% *W*_peak_ (*P* = 0.043), remaining reduced through to *W*_peak_. Concomitantly, forearm skin blood flow more than doubled, while forearm deep tissue O_2_ saturation decreased. When incremental exercise started shortly after constant load exercise (test 2), ${\dot{Q}}_{\mathrm{forearm}}$ was 2‐ to 3‐fold higher than during tests 1 and 3, whereas skin a‐vO_2_ diff was suppressed to a low level. Similar changes were observed during constant load exercise. In conclusion, skin perfusion increases during incremental exercise in the heat, concomitant to proportional reductions in oxygen extraction from the cutaneous circulation. Hence, contrary to the generally held view, skin perfusion remains elevated during maximal exercise and heat stress despite profound increases in sympathoadrenal activity.

## Abstract (highlights)

- **What is the central question of the study?**
  Do perfusion and blood oxygen in the non‐exercising limbs, including those of the skin circulation, decline markedly during high‐to‐maximal intensity exercise?
- **What is the main finding and its importance?**
  Blood flow in the non‐exercising limb increases, then stabilises, during incremental exercise in the heat, concomitant to core temperature‐related increases in skin perfusion and secondary reductions in skin arterial‐to‐venous oxygen difference. This compensatory haemodynamic response was similar during constant load exercise. These data challenge the premise that skin blood flow declines at near‐maximal dynamic exercise.

## 1 INTRODUCTION

Dynamic exercise of increasing intensity evokes a complex series of haemodynamic adjustments, which differ across and within tissues and organs of the human body (Bevegård & Shepherd, 1967; Joyner & Casey, 2015; Nobrega et al., 2014; Travers et al., 2022). At the regional and systemic level, active skeletal muscle and systemic blood flow increases progressively and then plateaus (Calbet et al., 2007; González‐Alonso et al., 2015; Hermansen et al., 1970; Mortensen et al., 2005; Thomas & Segal, 2004), whereas blood flow declines in the renal, splanchnic and brain circulations (Perko et al., 1998; Rowell, 1974; Rowell et al., 1964; Trangmar et al., 2017, 2014) and diverges in the cardiac, respiratory and inactive skeletal muscle circulations (Calbet et al., 2007; Mortensen et al., 2008; Vogiatzis et al., 2009). These varied responses in regional blood flow occur against a backdrop of an exponential increase in whole‐body sympathoadrenal activity (above moderate‐intensity exercise) that modulates local vascular tone in tissues and organs, primarily in relation to the local metabolic and thermoregulatory needs (Callister et al., 1994; Ichinose et al., 2008; Katayama & Saito, 2019; Kjaer et al., 1993; Perko et al., 1998; Rosenmeier et al., 2004; Rowell, 1974; Savard et al., 1987, 1989). While local mechanisms effectively override the prevailing vasoconstrictor tone in contracting‐skeletal‐muscle beds (Remensnyder et al., 1962), enabling substantial increases in blood flow, the vasculature of other non‐active tissue beds, including the non‐exercising skeletal muscle and overlying skin of limbs (e.g., the arms during lower‐limb exercise), might see their blood flow reduced (Laughlin et al., 2012) presumably due to pronounced elevations in sympathetic nerve activity and circulating vasoconstrictor substances (Bevegård & Shepherd, 1967; Kellogg et al., 1991a). Despite blood flow being widely studied, there remains conflicting data to support the idea that blood flow in the inactive limbs is suppressed during whole‐body exercise of increasing intensity.

Of the limited available data, total blood flow and vascular conductance in the resting limb (typically measured in the arm/forearm) have either been shown to decrease (Bevegård & Shepherd, 1966; Bishop et al., 1957; Blair et al., 1961; Johnson & Rowell, 1975; Zelis et al., 1969), or be unchanged (Calbet et al., 2007; González‐Alonso et al., 2015) or increase (Birk et al., 2012; Green, Cheetham, Reed, et al., 2002; Padilla et al., 2011; Simmons et al., 2011; Tanaka et al., 2006; Zelis et al., 1969) during low‐to‐moderate intensity exercise. These inconsistent findings might be largely a result of differences in the experimental design (e.g., exercise intensity, exercise duration and environmental conditions) and the methods used to interrogate forearm/arm blood flow (e.g. venous occlusion plethysmography, indirect estimations based on axillary venous oxygen saturation, thermodilution and Doppler ultrasound). Generally, the rest‐to‐exercise transition is associated with an initial reduction in inactive‐limb blood flow and vascular conductance (Green, Cheetham, Reed, et al., 2002; Padilla et al., 2011; Simmons et al., 2011). This initial vasoconstriction is reversed as exercise continues, with forearm blood flow and vascular conductance shown to increase over time (during sustained sub‐maximal exercise) (Birk et al., 2012; Padilla et al., 2011; Simmons et al., 2011). Although the majority of these studies were conducted in a thermoneutral environment, there is evidence linking the rise in local tissue and core temperature to increasing inactive‐limb blood flow and vascular conductance (Simmons et al., 2011). This supports a mechanistic role of thermosensitive pathways in the observed increase in inactive‐limb perfusion, where the local metabolic demand remains essentially unchanged. In respect to the influence of exercise intensity, only two studies report blood flow, oxygen extraction and ${\dot{V}}_{O_{2}}$ data at the heavy/severe and maximal exercise intensities in a thermoneutral environment (Calbet et al., 2007; González‐Alonso et al., 2015). In these studies, arm blood flow and ${\dot{V}}_{O_{2}}$ were similar to baseline values up to ∼85% of *W*_max_, increasing during the maximal exercise stage in parallel to a significant rise in arm oxygen extraction. The augmented arm ${\dot{V}}_{O_{2}}$ when approaching exhaustion suggests increased metabolic demand of the apparently inactive limb during leg exercise. However, it is possible that the arms in this multi‐study investigation were not completely at rest, as they were held above the head, with hands place on the crank handles of a second arm crank ergometer, gripping to stabilize torso movement during very intense upright two‐leg cycling exercise (Calbet et al., 2007; González‐Alonso et al., 2015). Hence, knowledge and understanding of the responses of the inactive limb tissues and skin to near‐to‐maximal aerobic exercise remain incomplete.

When looking at responses within limb tissues, available evidence suggests that the reduction in inactive limb blood flow is equally shared between the non‐exercising skeletal muscle (Blair et al., 1961; Johnson & Rowell, 1975; Zelis et al., 1969) and the overlying skin circulation (Johnson & Park, 1982; Taylor et al., 1984; Zelis et al., 1969). Based on the purported limitations to cardiovascular capacity during exhaustive exercise, where there is a widespread increase in sympathoadrenal (i.e. vasoconstrictor) activity, these observations have led to the hypothesis that further reductions in inactive limb and skin blood flow occur as exercise progresses to maximal intensities (Rowell, 1974; Wade & Bishop, 1962). This theory remains widely reported in comprehensive topical reviews to this day (Laughlin et al., 2012; Périard et al., 2021), despite being based on limited data obtained during submaximal exercise. Moreover, the originally estimated ∼75% decline in inactive tissue and skin blood flow during graded exercise (e.g., from ∼2 to 0.5 L/min), is unlikely to be realistic given that the postulated maximal cardiac output (25 L/min) in untrained individuals is markedly overestimated (i.e., ≥10 L/min) based on the referenced ${\dot{V}}_{O_{2}\max}$ data of ∼2 L/min (Wade & Bishop, 1962). The assumed substantial reduction in inactive‐limb and skin blood flow during exhaustive exercise has been postulated to occur when similar exercise is performed in hot environments (Périard et al., 2021; Rowell, 1974). This assumes that peripheral and systemic blood flow are reduced in hot environments (Rowell, 1974) and that the original inactive‐limb and skin haemodynamic data from thermoneutral environments reflect responses during exercise in the heat. However, skin blood flow and forearm vascular conductance increase profoundly during exercise in normothermic conditions (Birk et al., 2012; Green, Cheetham, Reed, et al., 2002; Padilla et al., 2011), and warm–hot environments (Bishop et al., 1957; González‐Alonso et al., 1998, González‐Alonso, Teller, et al., 1999; Johnson & Rowell, 1975; Kenney & Johnson, 1992; Ooue et al., 2008; Simmons et al., 2011; Taylor et al., 1988, 1984). Moreover, a greater systemic blood flow can still be achieved during the early stages of maximal aerobic exercise in hyperthermic compared to control conditions in trained humans (González‐Alonso & Calbet, 2003; González‐Alonso et al., 2004). Hence, there is, at present, no direct evidence to support the idea that skin blood flow declines at near‐maximal exercise intensities in the heat. Knowledge of the circulatory responses to incremental exercise in conditions of thermal hyperaemia would therefore provide further insight about the control of blood flow in non‐exercising limb tissue in conditions of profound increases in sympathoadrenal activity.

The aim of the present study, therefore, was to systematically investigate the haemodynamic and oxygenation responses of the inactive forearm during incremental and constant, submaximal two‐leg exercise, in the heat under normal and hyperaemic conditions. The semi‐recumbent cycling model was used to ensure that the arm remained inactive. A second maximal exercise bout was undertaken shortly after the 2‐h exercise bout to create a hyperaemic condition at onset of exercise compared to the maximal tests 1 and 3 and thereby gain further insight into the control of blood circulation during exercise in the heat. Based on the available literature, we hypothesized that inactive forearm blood flow, predominantly reflecting the skin circulation, would be reduced at high exercise intensities in the heat. Furthermore, we hypothesized that the reduced blood flow would be reflected in forearm blood oxygenation and a‐vO_2_ differences.

## 2 METHODS

### 2.1 Ethical approval

All procedures in the present studies were approved by the Brunel University of London Research Ethics Committee (RE07‐11 and 18290‐MHR‐Mar/2020‐24938‐1, 40326‐MHR‐Nov/2022‐42081‐2; 40326‐A‐Aug/2023‐46741‐3; 40326‐A‐Jan/2024‐49530‐1) and conformed to the ethical principles of the World Medical Association (*Declaration of Helsinki*), except for the pre‐registration of the study in a database.

### 2.2 Participants

Sixteen cyclists, classified as tier 2 and 3 (McKay et al., 2022), participated in two studies. In study 1, nine male cyclists (means ± SD; age: 29 ± 5 years, height: 184 ± 5 cm, body mass: 79 ± 9 kg, and ${\dot{V}}_{O_{2}\mathrm{peak}}$: 59 ± 7 mL kg^−1^ min^−1^) participated. In study 2, seven male cyclists (means ± SD; age: 33 ± 4 years, height: 180 ± 2 cm, body mass: 80 ± 5 kg, and ${\dot{V}}_{O_{2}\mathrm{peak}}$: 57 ± 2 mL kg^−1^ min^−1^) participated. The aims, study design, methodology and risks of participating were fully explained to participants prior to obtaining their informed and written consent to participate. All participants completed a health screening form to ensure that they were free of any known cardiovascular, metabolic or respiratory disease. Prior to each experimental trial, participants were instructed to avoid heavy exercise and alcohol intake for 24 h and caffeine consumption for 12 h and arrive at the laboratory post‐prandial and in a euhydrated state.

### 2.3 Overview

The present paper contains data collected in two studies. The first study (study 1) was conducted between 2012 and 2013 and explored the circulatory, thermal and brain haemodynamic and metabolic responses to incremental and constant exercise in the heat, with and without dehydration (Trangmar et al., 2015, 2014). The present paper is a retrospective analysis of previously unpublished forearm venous blood gas and metabolite data, from the euhydrated control trial of that study. A second study (study 2) was a new study, conducted between 2022 and 2024, conceived to investigate the forearm blood flow and oxygenation responses to a range of haemodynamic conditions, invoked by different two‐leg exercise intensities and durations, in a hot environment.

### 2.4 Experimental preparation and measurements

**FIGURE 1:**
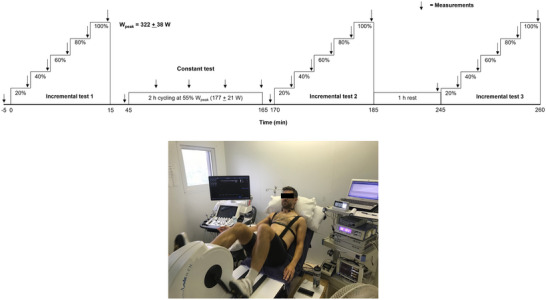
Schematic representation and image of the experimental protocol and an image depicting the experimental set‐up common to both studies. In both studies, the experimental trial consisted of three incremental cycle ergometer exercise tests of 3‐min stages at 20, 40, 60, 80 and 100% of *W*_peak_ (322 ± 38 W). Between tests 1 and 2, participants completed 2 h of constant load (55% *W*_peak_, 177 ± 21 W) cycling exercise. All exercise tests were completed in a hot environment (35°C, 50% relative humidity, fan cooling).

For the purposes of the present manuscript, both studies shared a similar experimental design. Participants visited the laboratory on two occasions, for one preliminary and one experimental visit. On the preliminary visit, participants completed an incremental exercise test to volitional exhaustion for the determination of ${\dot{V}}_{O_{2}\mathrm{peak}}$ and *W*_peak_. After an ∼15 min recovery period, participants cycled for ∼90 min, at 55% of their *W*_peak_ in the heat, to partly familiarize with the conditions of the experimental visit. At least 1 week later, participants returned for the experimental trial. The experimental trial comprised three incremental cycling exercise tests to volitional exhaustion, where the work rate was increased every 3 min to 20%, 40%, 60%, 80% and 100% of their peak work rate (*W*_peak_; 322 ± 38 W) (Figure 1). To further investigate the inactive‐limb's blood oxygenation response to dynamic exercise in the heat, participants completed 2 h of lower‐limb cycling exercise, at 55% of *W*_peak_ (177 ± 21 W), between incremental tests 1 and 2. Data for *W*_peak_ and 55% of *W*_peak_ listed above and in Figure 1 are pooled values from both studies. Cycling exercise was performed in the semi‐recumbent position on an electronically braked cycle ergometer (Lode Angio, Groningen, The Netherlands) (Figure 1), at a self‐selected pedal cadence between 70 and 90 rpm, with the arms supported in a resting position. All exercise bouts were performed in an environmental chamber set at 35°C (relative humidity: 50%; with fan cooling), and hydration was maintained throughout the exercise tests through the consumption of regular aliquots of cool carbohydrate–electrolyte solution (4.0 ± 0.6 L), according to the rate of fluid lost during the preliminary trials.

### 2.5 Blood sampling and analysis (study 1 only)

Venous blood samples were obtained from a catheter, inserted into the median cubital vein, in the anterograde direction (i.e. same direction of blood flow), of the non‐dominant forearm. Blood samples were drawn into pre‐heparinized syringes and rapidly analysed (ABL 800 FLEX, Radiometer, Copenhagen, Denmark) for haemoglobin (Hb), oxygen saturation ($S_{O_{2}}$), oxygen tension ($P_{O_{2}}$) and other blood metabolites. Blood gas variables were corrected for the corresponding core temperature values. The blood oxygen content was calculated from the saturation ($S_{O_{2}}$) and [Hb], that is [(1.34 [Hb] × $S_{O_{2}}$) + (0.003 × $P_{O_{2}}$)]. Samples were obtained at rest, at the end of each incremental exercise stage and every 30 min during constant load exercise. Arterial blood values were estimated based on the measured venous haemoglobin concentration and the arterial $S_{O_{2}}$ and $P_{O_{2}}$ values observed during the invasive trial previously reported (Trangmar et al., 2014).

### 2.6 Non‐exercising limb haemodynamics (study 2 only)

Blood flow to the non‐exercising limb (${\dot{Q}}_{\mathrm{forearm}}$) was measured in the brachial artery using duplex vascular ultrasound (Vivid E90 Dimension, GE Healthcare, Chalfont St. Giles, UK), equipped with a 9 MHz linear array transducer. Average diameter and flow velocity profiles were made from >12 cardiac cycles to attenuate respiration artefacts. The Doppler gate was placed in the centre of the vessel lumen, in the direction of the blood flow, and adjusted to cover its width, while the insonation angle was maintained at <60°. Mean flow velocity profiles were traced automatically and analysed offline for determination of mean blood flow velocity (TAM V) (EchoPAC BT12, Version: 112, GE Healthcare, Horton, Norway). Blood flow (in ml/min) was then calculated using mean flow velocity × cross‐sectional area (CSA; where CSA = π × (mean diameter/2)^2^, and blood flow = time averaged mean flow velocity × CSA × 60). Mean vessel diameter was measured manually, by way of digital callipers, and calculated as a weighted average of vessel diameter across the cardiac cycle. The approach to calculating blood flow was consistent with our previous work (Trangmar et al., 2015, 2014) and in line with the general consensus (Schöning et al., 1994)

### 2.7 Additional cardiovascular, temperature and muscle activity measures

In both studies, heart rate (HR) was obtained by wireless telemetry (Polar Electro, Kempele, Finland). Core temperature was assessed using an ingestible telemetry pill (HQ, Palmetto, FL, USA; study 1) and a rectal thermocouple, self‐inserted 15 cm beyond the anal sphincter (PhysiTemp, Clifton, NJ; study 2). In study 2 only, forearm skin blood flow (${\dot{Q}}_{\mathrm{Skin}}$), from a normothermic baseline, was measured by laser Doppler flowmetry (Periflux 4001; Jarfalla, Sweden) via a 780‐nm wavelength single‐point laser Doppler probe (408, Periflux) secured to the surface of the skin, reported as perfusion units (PU). Forearm muscle oxygen saturation (r $S_{O_{2}}$ %) was assessed using near‐infrared spectroscopy (NIRS; INVOS, Somanetics, Troy, MI, USA). The INVOS oximetry sensors allowed for continuous assessment of forearm tissue oxygenation by way of infrared light, emitted at wavelengths of 730 and 810 nm, and two independent detectors at 3 and 4 cm from the light source, typically measuring to a depth of 20–25 mm below the skin surface. Skin temperature of the forearm (*T*_sk_) was measured using a wired thermocouple (PhysiTemp T‐204A; Clifton, NJ, USA), attached to a thermocouple meter (TC‐2000 Type‐T, Sable Systems, Las Vegas, NV, USA).

### 2.8 Data analysis

Values are expressed as the mean ± SD. Each haemodynamic and blood gas parameter was assessed over time/exercise intensity using a one‐way repeated‐measures ANOVA. Where appropriate, two‐way repeated‐measures ANOVA was used to compare responses during incremental exercise. Where a significant main effect was found, pairwise comparisons were made using the Holm–Bonferroni *post hoc* procedure. Statistical significance was set at *P* <0.05 and all analyses were made using IBM SPSS Statistics (Version 29, IBM Corp., Armonk, NY, USA).

## 3 RESULTS

### 3.1 Blood oxygen content and arterio‐venous oxygen difference in the non‐exercising forearm during incremental and constant‐load exercise

**FIGURE 2:**
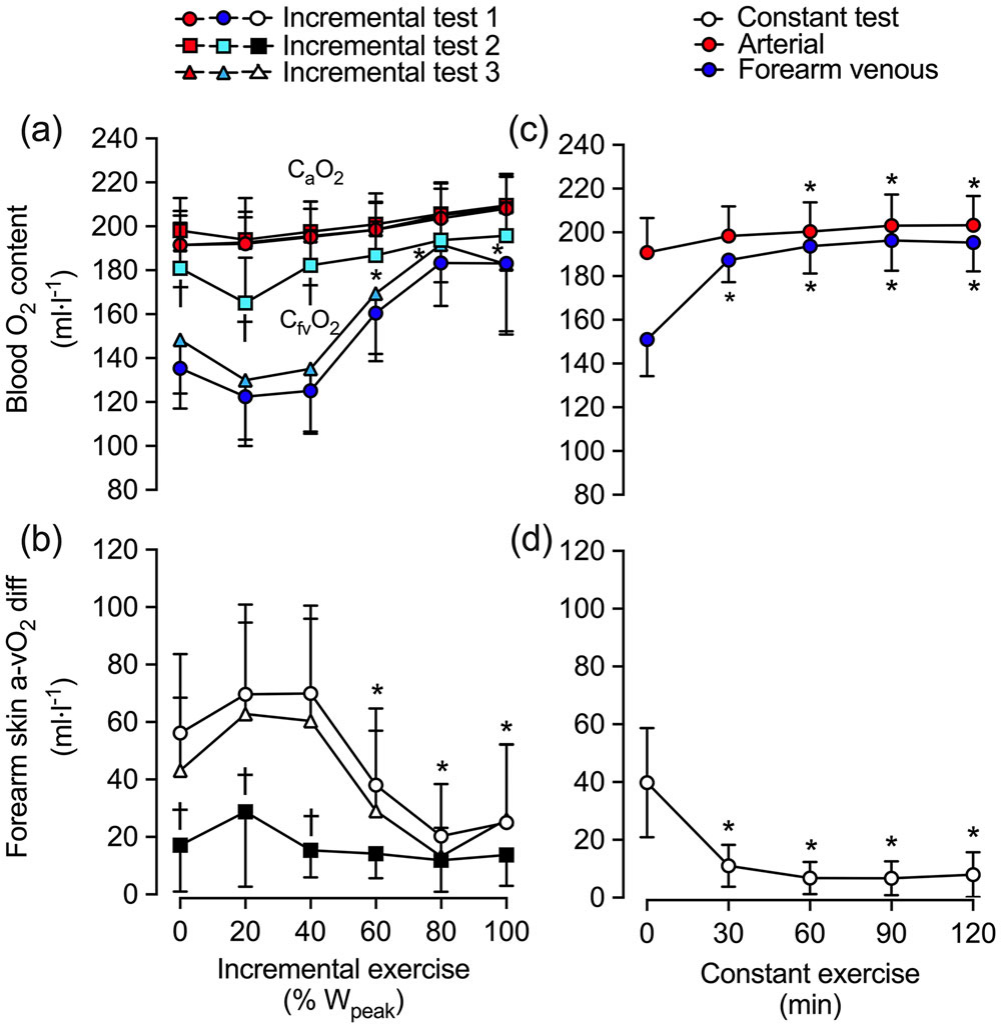
Arterial (C_a_O_2_) and forearm venous (C_fv_O_2_) blood O_2_ content, and forearm skin a‐vO_2_ difference during repeated incremental (a, b) and constant, sub‐maximal (c, d) exercise in the heat. Values are means ± SD for nine participants. *Differences from rest, *P* <0.05; ^†^differences from incremental tests 1 and 3, *P* <0.05.

During incremental exercise, arterial oxygen content increased with exercise intensity in all three incremental tests, with an average peak value ∼8% higher at peak exercise compared to rest (209 ± 14 vs. 191 ± 15 mL/L, *P* <0.0001; Figure 2a). Forearm venous oxygen content remained stable in early exercise before increasing thereafter, in all incremental tests, to ∼80% *W*_peak_ (peak vs. rest; 187 ± 22 vs. 155 ± 12 mL/L, *P* <0.0001). Forearm skin a‐vO_2_ difference response was similar in incremental exercise tests 1 and 3 but differed in test 2. In tests 1 and 3, a‐vO_2_ difference remained stable in early exercise, before decreasing from 66 ± 29 mL/L, at 20% *W*_peak_, to a nadir of ∼17 ± 13 mL/L at 80% *W*_peak_ (Figure 2b, *P* = 0.043). In test 2, a‐vO_2_ difference was elevated compared to tests 1 and 3, and remained at a high level, similar to values seen at peak exercise in tests 1 and 3, throughout incremental exercise (17 ± 14 mL/L, *P* = 0.660; Figure 2b).

During constant load exercise, there was an overall increase in arterial oxygen content compared to resting values (from 191 ± 16 to 201 ± 13 mL/L, *P* <0.0001; Figure 2c). No changes occurred between 30 and 60 min, before increasing at 90 (*P* = 0.014) and 120 min (*P* = 0.008) (both vs. 30 min value). Forearm venous oxygen content increased from rest to 30 min of constant load exercise (*P* <0.0001) and thereafter remained stable until the end of exercise (Figure 2c). Forearm a‐vO_2_ difference decreased from rest to mid‐way (i.e. up to 60 min) through constant load exercise (40 ± 19 vs. 9 ± 6 mL/L; *P* <0.0001), before plateauing thereafter (Figure 2d).

### 3.2 Forearm blood flow, skin blood flow and tissue O_2_ saturation in the non‐exercising forearm, and rectal and skin temperatures during incremental and constant load exercise

**FIGURE 3:**
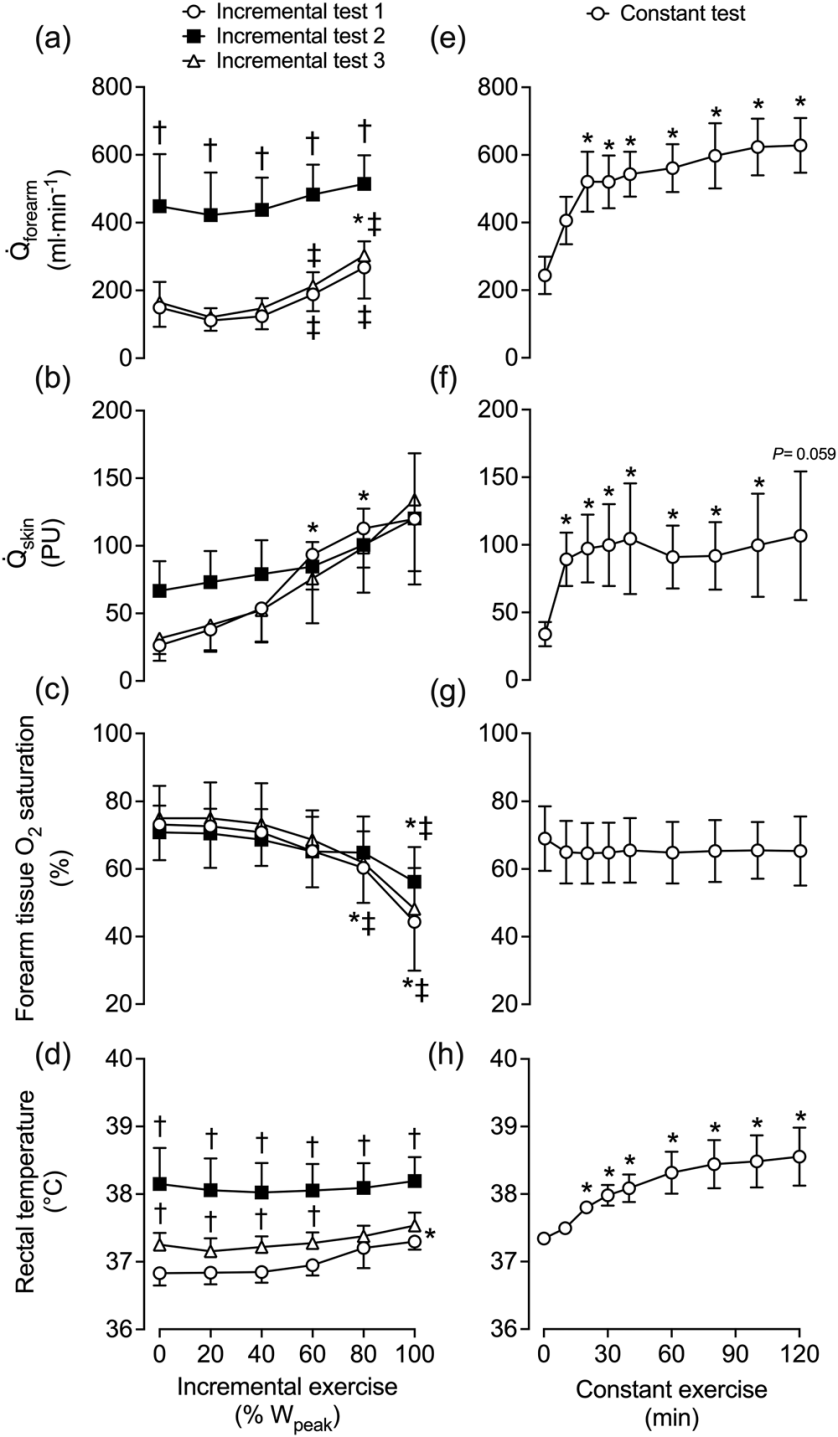
Forearm blood flow, forearm skin blood flow, forearm tissue O_2_ saturation % and rectal temperature during repeated incremental (a–d) and constant, sub‐maximal (e–h) exercise in the heat. Values are means ± SD for nine participants. *Differences from rest, *P* <0.05; ^‡^differences from sub‐maximal (pooled average of 20% and 40% *W*_peak_ values) exercise, *P* <0.05; ^†^differences from tests 1 and 3, *P* <0.05.

Forearm blood flow (${\dot{Q}}_{\mathrm{forearm}}$) was stable from rest to ∼40% *W*_peak_ during incremental tests 1 and 3, before increasing to a peak of 285 ± 52 mL/min at 80% *W*_peak_ (*P* <0.001; Figure 3a). The increase in ${\dot{Q}}_{\mathrm{forearm}}$ was brought about by increasing blood flow velocity, as vessel diameter was unchanged in all incremental exercise tests. In test 2, which was performed ∼5 min after 2 h of constant load cycling exercise, ${\dot{Q}}_{\mathrm{forearm}}$ was high at rest (∼449 ± 153 mL/min) and remained high throughout incremental exercise, peaking at 514 ± 84 mL/min (*P* = 0.270). In test 1, skin blood flow (${\dot{Q}}_{\mathrm{skin}}$) progressively increased from rest to 80% *W*_peak_ (26 ± 6 vs. 113 ± 29 PU; *P* = 0.014), before plateauing through to *W*_peak_ (Figure 3b). A similar response was observed in test 3 (albeit ${\dot{Q}}_{\mathrm{skin}}$ continued to increase through to *W*_peak_). In test 2, ${\dot{Q}}_{\mathrm{skin}}$ from rest to 40% *W*_peak_ was unchanged, before increasing between 60% and 80% *W*_peak_ (93 ± 21 vs. 73 ± 21 PU; *P* = 0.041). ${\dot{Q}}_{\mathrm{skin}}$ at *W*_peak_ was higher at *W*_peak_ in test 2 versus all other exercise intensities (*P* ≈ 0.025; Figure 3b). Forearm tissue O_2_ saturation was unchanged from rest to sub‐maximal exercise intensities, before declining in all three incremental exercise tests beyond 80% *W*_peak_ (*P* <0.0001; Figure 3c). Rectal temperature increased during incremental exercise in tests 1 and 3 (*P* <0.0001) but was maintained to a high level throughout test 2 (Figure 3d; *P* = 0.260). Forearm skin temperature was unchanged at all exercise intensities, among all incremental tests (∼34.5 ± 1°C; *P* = 0.248).

During constant load exercise, ${\dot{Q}}_{\mathrm{forearm}}$ increased from rest (∼218 ± 52 mL/min) to 30 min (∼521 ± 89 mL/min) (*P* <0.0001; Figure 3e), thereafter remaining at this high level, before increasing at the end of constant load exercise (∼629 ± 81 mL/min; vs. *P* = 0.011 vs. 40 min value). The increase in blood flow was accomplished by increased blood velocity (∼+166% vs. baseline; *P* <0.0001) and brachial artery diameter (∼ +10% vs. baseline; *P* = 0.008). ${\dot{Q}}_{\mathrm{skin}}$ increased from rest to 10 min (34 ± 9 vs. 89 ± 20 PU; *P* = 0.005) and was thereafter unchanged throughout constant load exercise (Figure 3f). Contrastingly, there was no change in forearm tissue O_2_ saturation through constant load exercise (Figure 3g). Rectal temperature progressively increased from rest (37.3 ± 0.1°C) to 80 min (38.4 ± 0.4°C; *P* <0.0001), before plateauing through to the end of constant load exercise (Figure 3h). Forearm skin temperature was elevated at 40 min compared to early exercise (35.1 ± 0.4 vs. 34.7 ± 0.6°C; *P* = 0.010), remaining stable until the end of constant load exercise.

### 3.3 Venous blood gas and metabolite responses in the non‐exercising forearm during incremental cycling exercise and constant load exercise

Venous non‐exercising forearm pH, haemoglobin and blood gases during the three incremental exercise tests, along with statistical results, are presented in Table 1. Most venous blood gases (except Hb) were unchanged from rest to 40% *W*_peak_. At *W*_peak_, compared to 80% *W*_peak_, pH was reduced in incremental tests 1 and 3, and Hb increased in all incremental tests. Fractional oxyhaemoglobin (${F_{O_{2}}}_{\mathrm{Hb}}$_%_), fractional methaemoglobin (*F*_MetHb%_), and fractional carboxyhaemoglobin (*F*_COHb%_) were essentially unchanged throughout incremental exercise. $P_{CO_{2}}$ began to fall from ∼60% to 80% *W*_peak_, while $P_{O_{2}}$ and $S_{O_{2}}$ increased in incremental tests 1 and 3, but owing to the prior bout of constant sub‐maximal exercise, no changes were observed in incremental test 2 (Table 1).

**TABLE 1:** Forearm venous blood gas responses in the non‐exercising forearm during incremental cycling exercise.

		Exercise intensity (%*W*_peak_)						
	Rest	20	40	60	80	100	Factor	*P*
pH								
Incremental test 1	7.38 ± 0.03	7.36 ± 0.03	7.35 ± 0.03	7.35 ± 0.04	7.34 ± 0.04	7.30 ± 0.05^a^, ^b^	Test	0.006
Incremental test 2	7.39 ± 0.03	7.39 ± 0.03	7.39 ± 0.05	7.38 ± 0.07	7.37 ± 0.07	7.26 ± 0.14	Intensity	<0.001
Incremental test 3	7.40 ± 0.04	7.40 ± 0.04	7.41 ± 0.04	7.42 ± 0.04	7.43 ± 0.04	7.39 ± 0.05^b^	Interaction	<0.001
Hb (g L^−1^)								
Incremental test 1	141 ± 10	142 ± 9	144 ± 10^a^, ^b^	147 ± 9^a^	151 ± 10^a^, ^b^	154 ± 11^a^, ^b^	Test	0.032
Incremental test 2	146 ± 11	143 ± 14	146 ± 10	149 ± 11	152 ± 11^a^, ^b^	155 ± 11^a^, ^b^	Intensity	<0.001
Incremental test 3	141 ± 12	142 ± 10	144 ± 12^a^	147 ± 10^a^	152 ± 11^a^, ^b^	154 ± 10^a^, ^b^	Interaction	0.145
${F_{O_{2}}}_{\mathrm{Hb}}$ (%)								
Incremental test 1	69 ± 13	62 ± 14	63 ± 14	78 ± 13^b^	87 ± 8^a^	84 ± 13	Test	<0.001
Incremental test 2	88 ± 8	83 ± 12	89 ± 4	90 ± 4	91 ± 5	90 ± 5	Intensity	<0.001
Incremental test 3	75 ± 12	65 ± 15	67 ± 17	82 ± 14^b^	90 ± 5	88 ± 6	Interaction	<0.001
FMetHb (%)								
Incremental test 1	0.7 ± 0.1	0.7 ± 0.1	0.7 ± 0.1	0.7 ± 0.1	0.7 ± 0.1	0.7 ± 0.1	Test	0.002
Incremental test 2	0.7 ± 0.1	0.7 ± 0.2	0.7 ± 0.1	0.6 ± 0.1	0.6 ± 0.1	0.6 ± 0.2	Intensity	0.160
Incremental test 3	0.7 ± 0.1	0.8 ± 0.1	0.7 ± 0.1	0.7 ± 0.1	0.7 ± 0.1	0.7 ± 0.1	Interaction	0.581
FCOHb (%)								
Incremental test 1	1.4 ± 0.2	1.3 ± 0.2	1.3 ± 0.2	1.4 ± 0.2	1.4 ± 0.2	1.3 ± 0.2	Test	0.279
Incremental test 2	1.3 ± 0.2	1.3 ± 0.2	1.4 ± 0.2	1.4 ± 0.2	1.4 ± 0.2	1.3 ± 0.2	Intensity	0.007
Incremental test 3	1.3 ± 0.3	1.1 ± 0.2	1.2 ± 0.2	1.3 ± 0.2	1.4 ± 0.2	1.3 ± 0.2	Interaction	0.005
$P_{CO_{2}}$ (mmHg)								
Incremental test 1	50 ± 5	51 ± 5	52 ± 5	46 ± 8	45 ± 5^a^	42 ± 7^a^, ^b^	Test	<0.001
Incremental test 2	44 ± 3	44 ± 4	43 ± 4	42 ± 4^a^, ^b^	40 ± 4^a^, ^b^	38 ± 4^a^, ^b^	Intensity	<0.001
Incremental test 3	45 ± 6	46 ± 7	48 ± 4	46 ± 5^b^	42 ± 4^b^	42 ± 6	Interaction	0.062
$P_{O_{2}}$ (mmHg)								
Incremental test 1	42 ± 15	37 ± 10	38 ± 9	51 ± 11^b^	65 ± 14^a^, ^b^	66 ± 19^a^	Test	0.006
Incremental test 2	67 ± 15	58 ± 16	65 ± 10	66 ± 10	73 ± 14	72 ± 13	Intensity	0.003
Incremental test 3	50 ± 20	43 ± 17	43 ± 15	56 ± 13^b^	69 ± 12^a^, ^b^	64 ± 17	Interaction	<0.001
$S_{O_{2}}$ (%)								
Incremental test 1	67 ± 9	63 ± 15	64 ± 14	80 ± 13^a^, ^b^	89 ± 8^a^, ^b^	86 ± 13^a^	Test	<0.001
Incremental test 2	90 ± 8	84 ± 12	91 ± 5	92 ± 4	93 ± 5	92 ± 5	Intensity	<0.001
Incremental test 3	77 ± 13	67 ± 16^a^	69 ± 17	84 ± 14^b^	92 ± 5^a^, ^b^	86 ± 13^a^, ^b^	Interaction	<0.001

Values are means ± SD for nine participants. *P* values were determined using two‐way ANOVA with repeated measures and the Holm‐Bonferroni post hoc procedure. pH, haemoglobin (Hb), fractional oxyhaemoglobin (${F_{O_{2}}}_{\mathrm{Hb}}$%), fractional methaemoglobin (*F*_MetHb_%), fractional carboxyhaemoglobin (*F*_COHb_%), partial pressures of carbon dioxide ($P_{CO_{2}}$) and oxygen ($P_{O_{2}}$), and oxygen saturation ($S_{O_{2}}$%). pH, $P_{CO_{2}}$ and $P_{O_{2}}$ denote temperature corrected values.

^a^ Different versus rest.

^b^ Different versus previous intensity.

Venous non‐exercising forearm metabolite responses during the three incremental exercise tests are presented in Table 2. Bicarbonate ([HCO_3_^−^]) was unchanged from rest to 80% *W*_peak_ in all incremental tests, before declining at *W*_peak_ (Table 2). This reduction at *W*_peak_ was mirrored by a decline in acid–base excess, and a substantial increase in venous blood lactate. Venous blood glucose was unchanged from rest to *W*_peak_ in incremental test 1 but fell beyond ∼40% *W*_peak_ in maximal test 2 and 3. Potassium (K^+^) and sodium (Na^+^) increased in all three incremental tests beyond 40% *W*_peak_, and chlorine (Cl^−^) was unchanged in all three incremental tests (except for increasing at *W*_peak_ in incremental test 2).

**TABLE 2:** Forearm venous acid‐based balance, metabolite and electrolyte responses in the non‐exercising forearm during incremental cycling exercise.

		Exercise intensity (%*W*_peak_)						
	Rest	20	40	60	80	100	Factor	*P*
[HCO_3_^−^] (mmol L^−1^)								
Incremental test 1	26.2 ± 1.5	25.6 ± 2.2	25.2 ± 1.9	23.5 ± 3.9	22.7 ± 2.0^a^	19.4 ± 1.9^a^, ^b^	Test	0.003
Incremental test 2	25.7 ± 1.5	25.1 ± 2.4	25.4 ± 2.5	24.8 ± 3.3	23.6 ± 3.2^a^	21.8 ± 2.9^a^, ^b^	Intensity	<0.001
Incremental test 3	25.6 ± 2.1	27.0 ± 2.1	27.4 ± 1.5	28.0 ± 1.7	27.1 ± 2.1	24.2 ± 2.2^b^	Interaction	<0.001
ABE (mmol L^−1^)								
Incremental test 1	3.6 ± 1.9	3.2 ± 2.7	2.8 ± 2.2	−0.2 ± 5	−1.4 ± 2.5^a^	−5.7 ± 2.9^a^, ^b^	Test	0.001
Incremental test 2	1.8 ± 1.6	1.3 ± 2.7	1.5 ± 2.8	0.5 ± 3.7	−1.1 ± 3.7^a^, ^b^	−5.2 ± 5^a^, ^b^	Intensity	<0.001
Incremental test 3	2.3 ± 2.6	4.3 ± 2.5^a^	4.9 ± 1.2^a^	4.8 ± 1.6^a^	3.3 ± 2.4^b^	0.5 ± 2.2^b^	Interaction	<0.001
Lactate (mmol L^−1^)								
Incremental test 1	1.0 ± 0.4	1.0 ± 0.3	1.3 ± 0.3^a^, ^b^	1.9 ± 0.4^a^, ^b^	4.3 ± 1.0^a^, ^b^	8.7 ± 1.8^a^, ^b^	Test	0.503
Incremental test 2	2.0 ± 0.7	1.8 ± 0.5	1.8 ± 0.5	1.9 ± 0.5^b^	3.5 ± 0.9^a^, ^b^	6.8 ± 1.6^a^, ^b^	Intensity	<0.001
Incremental test 3	2.1 ± 0.5	2.0 ± 0.5	2.0 ± 0.5	2.1 ± 0.3	3.4 ± 0.5^a^, ^b^	7.3 ± 1.0^a^, ^b^	Interaction	<0.001
Glucose (mmol L^−1^)								
Incremental test 1	5.4 ± 0.6	5.2 ± 0.4	5.1 ± 0.3	5.2 ± 0.5	5.2 ± 0.7	5.0 ± 0.9	Test	<0.001
Incremental test 2	7.1 ± 0.7	6.6 ± 0.9	6.6 ± 0.8^*^	6.0 ± 0.7^a^, ^b^	5.2 ± 0.7^a^, ^b^	4.6 ± 0.6^a^, ^b^	Intensity	<0.001
Incremental test 3	6.1 ± 0.9	5.7 ± 0.8^a^	5.3 ± 0.8^a^, ^b^	4.6 ± 0.6^a^, ^b^	4.0 ± 0.4^a^, ^b^	3.6 ± 0.2^a^, ^b^	Interaction	<0.001
K^+^ (mmol L^−1^)								
Incremental test 1	4.0 ± 0.3	4.3 ± 0.3^a^	4.4 ± 0.4^a^, ^b^	4.7 ± 0.3^a^, ^b^	5.3 ± 0.5^a^, ^b^	6.0 ± 0.7^a^, ^b^	Test	0.011
Incremental test 2	4.2 ± 0.2	4.1 ± 0.4	4.5 ± 0.2^a^, ^b^	4.8 ± 0.2^a^, ^b^	5.2 ± 0.3^a^, ^b^	5.6 ± 0.3^a^, ^b^	Intensity	<0.001
Incremental test 3	3.8 ± 0.2	3.9 ± 0.2^a^	4.0 ± 0.2^a^	4.4 ± 0.2^a^ ^b^	4.8 ± 0.2^a^ ^b^	5.3 ± 0.2^a^, ^b^	Interaction	<0.001
Na^+^ (mmol L^−1^)								
Incremental test 1	137 ± 4	138 ± 3	139 ± 3^a^ ^b^	140 ± 3^a^ ^b^	143 ± 3^a^, ^b^	145 ± 4^a^ ^b^	Test	0.060
Incremental test 2	136 ± 5	136 ± 6	137 ± 4	138 ± 4^a^	140 ± 3^a^, ^b^	141 ± 2^a^	Intensity	0.041
Incremental test 3	136 ± 5	136 ± 5	137 ± 6	138 ± 6^a^, ^b^	139 ± 6^a^	141 ± 7^a^, ^b^	Interaction	0.024
Cl^−^ (mmol L^−1^)								
Incremental test 1	106 ± 3	105 ± 4	105 ± 5	109 ± 8	108 ± 5	110 ± 5	Test	0.112
Incremental test 2	107 ± 3	108 ± 5	107 ± 3	108 ± 3	108 ± 2	110 ± 3^a^, ^b^	Intensity	<0.001
Incremental test 3	106 ± 2	106 ± 2	106 ± 3	107 ± 3	108 ± 3	109 ± 4	Interaction	0.018

Values are means ± SD for nine participants. *P* values were determined using two‐way ANOVA with repeated measures and the Holm‐Bonferroni post hoc procedure. Bicarbonate ([HCO3^−^]), acid–base excess (ABE), lactate, glucose, potassium (K^+^), sodium (Na^+^) (*n *= 7) and chlorine (Cl^−^) (*n* = 6).

^a^ Different versus rest.

^b^ Different versus previous intensity.

Venous non‐exercising forearm blood gases and metabolite responses to 120 min of constant sub‐maximal exercise are presented in Table 3. Venous pH was elevated at 90 min compared to rest (7.40 ± 0.02 vs. 7.36 ± 0.04; *P* = 0.036), with no other differences at any other time points. Hb, ${F_{O_{2}}}_{\mathrm{Hb}}$, $P_{O_{2}}$ and $S_{O_{2}}$ increased from rest to 30 min (Table 3), before remaining at a similarly high value throughout the remainder of exercise. No other alterations in venous blood gases were observed. Of the blood metabolites, only blood lactate and K^+^ were altered during constant load exercise, where blood lactate fell (4.4 ± 1.6 vs. 2.5 ± 0.9 mmol/L; *P* <0.0001) and K^+^ increased (4.2 ± 0.4 vs. 4.9 ± 0.3 mmol/L; *P* = 0.001) from rest to 30 min, before remaining stable thereafter until the end of exercise.

**TABLE 3:** Forearm venous blood gas, acid–base balance, metabolite and electrolyte responses in the non‐exercising forearm during constant load cycling exercise.

		Constant submaximal exercise time (min)				
	Rest	30	60	90	120	*P*
pH	7.36 ± 0.04	7.38 ± 0.02	7.39 ± 0.04	7.40 ± 0.02^a^	7.39 ± 0.04	0.046
Hb (g L^−1^)	140 ± 11	147 ± 10^a^	148 ± 10^a^	150 ± 11^a^	150 ± 10^a^	<0.001
${F_{O_{2}}}_{\mathrm{Hb}}$ (%)	77 ± 9	91 ± 3^a^	93 ± 3^a^	93 ± 3^a^	93 ± 3^a^	<0.001
*F*_MetHb_ (%)	0.7 ± 0.1	0.7 ± 0.1	0.7 ± 0.2	0.6 ± 0.1	0.7 ± 0.1	0.012
*F*_COHb_ (%)	1.4 ± 0.2	1.4 ± 0.3	1.4 ± 0.3	1.4 ± 0.2	1.4 ± 0.3	0.885
$P_{CO_{2}}$ (mmHg)	42 ± 7	42 ± 4	42 ± 3	41 ± 3	42 ± 3	0.763
$P_{O_{2}}$ (mmHg)	47 ± 14	72 ± 11^a^	82 ± 14^a^	79 ± 9^a^	79 ± 12^a^	<0.001
$S_{O_{2}}$ (%)	79 ± 10	93 ± 4^a^	95 ± 3^a^	95 ± 3^a^	94 ± 4^a^	<0.001
[HCO_3_^−^] (mmol L^−1^)	23.2 ± 2.3	24.1 ± 1.6	24.9 ± 2.9	25.3 ± 1.6	25.3 ± 2.5	0.120
ABE (mmol L^−1^)	−0.6 ± 3.1	−0.2 ± 2.1	0.5 ± 3.5	0.9 ± 2.0	1.0 ± 3.1	0.350
Lactate (mmol L^−1^)	4.4 ± 1.6	2.5 ± 0.9^a^	2.6 ± 0.9^a^	2.4 ± 0.9^a^	2.0 ± 0.8^a^	0.001
Glucose (mmol L^−1^)	6.1 ± 1.0	5.0 ± 1.2	5.6 ± 0.4	5.5 ± 0.4	5.2 ± 0.8	0.077
K^+^ (mmol L^−1^)	4.2 ± 0.4	4.9 ± 0.3^a^	5.0 ± 0.5^a^	4.9 ± 0.3^a^	5.0 ± 0.4^a^	0.001
Na^+^ (mmol L^−1^)	139 ± 6	139 ± 3	137 ± 5	139 ± 3	139 ± 2	0.528
Cl^−^ (mmol L^−1^)	108 ± 5	109 ± 1	112 ± 6	109 ± 3	108 ± 3	0.820

Values are means ± SD for nine participants. *P* values were determined using one‐way ANOVA with repeated measures and the Holm‐Bonferroni post hoc procedure. pH, haemoglobin (Hb), fractional oxyhaemoglobin (${F_{O_{2}}}_{\mathrm{Hb}}$%), fractional methaemoglobin (*F*_MetHb%_), fractional carboxyhaemoglobin (*F*_COHb%_), partial pressures of carbon dioxide ($P_{CO_{2}}$) and oxygen ($P_{O_{2}}$), oxygen saturation ($S_{O_{2}}$%), bicarbonate ([HCO3^−^]), acid–base excess (ABE), lactate, glucose, potassium (K^+^), sodium (Na^+^) (*n* = 7) and chlorine (Cl^−^) (*n* = 6). pH, $P_{CO_{2}}$ and $P_{O_{2}}$ denote temperature corrected values.

^a^ Different versus rest.

## 4 DISCUSSION

The aim of the present study was to provide direct evidence to support or refute the prevailing hypothesis that non‐exercising limb and skin blood flow is markedly reduced at exercise intensities close to or eliciting aerobic capacity. We found that forearm blood flow, forearm blood oxygenation and forearm skin perfusion increased when incremental exercise exceeded ∼40% *W*_peak_, remaining at a high level through to volitional exhaustion. In contrast, forearm skin arterial‐to‐venous oxygen difference was substantially reduced at near‐maximal exercise intensities. Internal body hyperthermia, invoked prior to incremental exercise by a preceding bout of prolonged constant exercise, elevated forearm and skin blood flow by 2‐ to 3‐fold at baseline and it remained high throughout incremental exercise. The increase in forearm (total) and skin blood flow at rest with hyperthermia, and at high exercise intensities in each of the incremental tests, was accompanied by proportional increases in skin perfusion and reductions in forearm skin a‐vO_2_ diff, such that the estimated forearm ${\dot{V}}_{O_{2}}$ was seemingly maintained. This points to the forearm remaining inactive during cycling exercise. Collectively, the present findings support that the increased or elevated total forearm blood flow during maximal aerobic exercise and heat stress reflects an enhanced rather than reduced skin circulation. This refutes the widely held view that cutaneous blood flow declines markedly during dynamic exercise of increasing intensity.

### 4.1 Non‐exercising limb haemodynamics and oxygenation

During the initial stages of incremental exercise in the heat, no changes in ${\dot{Q}}_{\mathrm{forearm}}$ or ${\dot{Q}}_{\mathrm{skin}}$ were observed when compared to resting baseline values (Figure 2). This finding agrees with previous observations, where inactive‐forearm blood flow is either unchanged or reduced on the rest‐to‐exercise transition (Bevegård & Shepherd, 1966; Bishop et al., 1957; Blair et al., 1961; Green, Cheetham, Reed, et al., 2002; Padilla et al., 2011; Simmons et al., 2011). The unchanged or reduced blood flow likely reflects downstream, sympathetically mediated vasoconstriction, predominantly in the vasculature of the inactive skeletal muscle where the majority of resting forearm blood flow is directed (Cooper et al., 1955; Simmons et al., 2011), and evidenced by reductions in forearm vascular conductance on the initiation of leg cycling exercise (Birk et al., 2012; Padilla et al., 2011; Simmons et al., 2011). Our estimates of forearm vascular conductance (not presented) based on arterial blood pressure and forearm blood flow data from different individuals are supportive of this idea.

**FIGURE 4:**
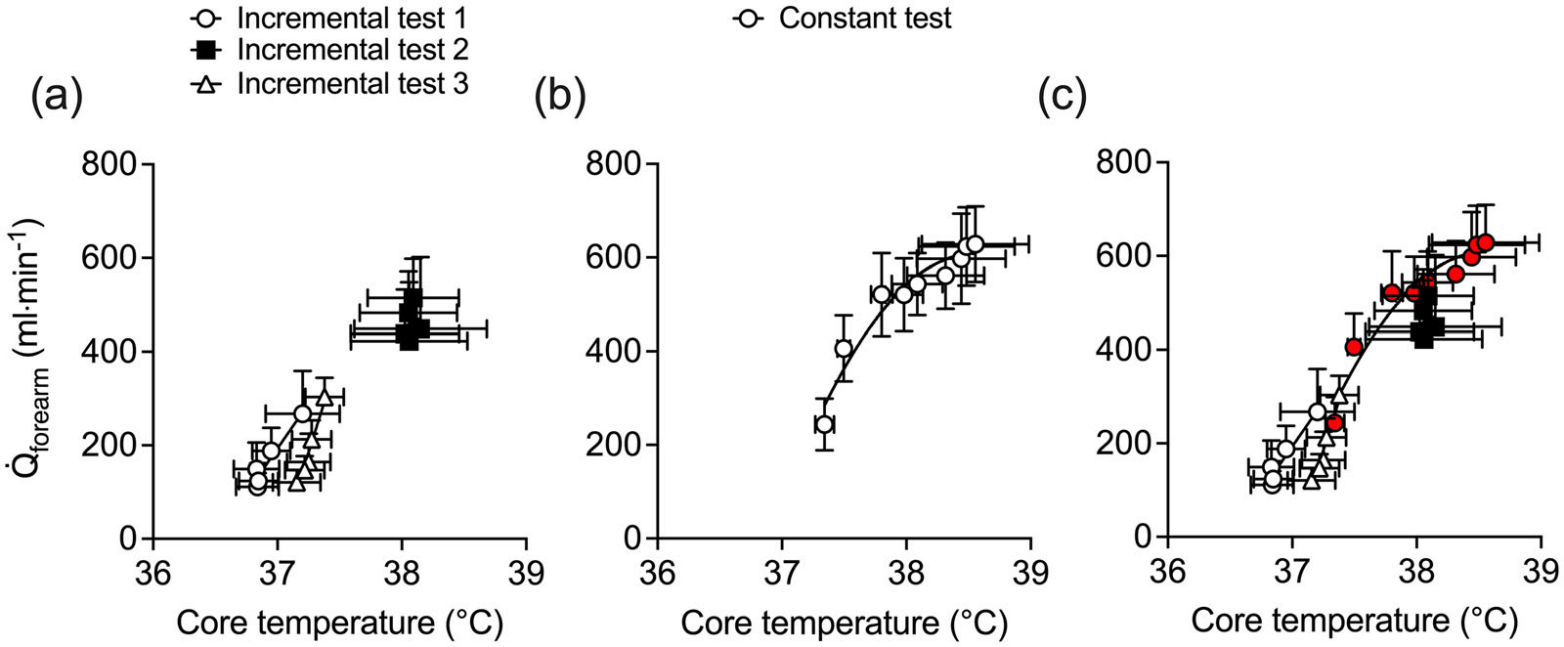
Relationship between ${\dot{Q}}_{\mathrm{forearm}}$ and core temperature during the repeated incremental (a) and prolonged, constant submaximal (b) exercise in the heat, and in both exercise conditions collectively (c). Values are means ± SD for nine participants.

As incremental exercise progressed beyond ∼40% *W*_peak_, both ${\dot{Q}}_{\mathrm{forearm}}$ and ${\dot{Q}}_{\mathrm{skin}}$ increased substantially (∼2‐ to 3‐fold). These findings extend previous observations of a stable, before increasing, ${\dot{Q}}_{\mathrm{forearm}}$ during low‐to‐moderate intensity exercise performed in normothermic conditions (Blair et al., 1961; Calbet et al., 2007; González‐Alonso et al., 2015; Green, Cheetham, Reed, et al., 2002; Johnson & Rowell, 1975; Ooue et al., 2008; Tanaka et al., 2006). We hypothesized that beyond submaximal exercise intensities, through to volitional exhaustion, ${\dot{Q}}_{\mathrm{forearm}}$ and ${\dot{Q}}_{\mathrm{skin}}$ would be reduced back to baseline values. This hypothesis was based on classical estimations of the distribution of whole‐body blood flow during incremental exercise in a thermoneutral environment (Rowell, 1974; Wade & Bishop, 1962), which are still depicted in recent/current literature (Laughlin et al., 2012; Périard et al., 2021). However, contrary to these estimations, we observed ${\dot{Q}}_{\mathrm{forearm}}$ and ${\dot{Q}}_{\mathrm{skin}}$ to increase through to ∼60–80% *W*_max_, and thereafter they remained high through to *W*_peak_ (Figure 2). These data indicate that the tissues of the non‐exercising forearm remain well‐perfused, despite the many fold increases in sympathoadrenal activity during strenuous exercise (Callister et al., 1994; Ichinose et al., 2008; Katayama & Saito, 2019; Taylor et al., 1992; Trangmar et al., 2017, 2014). The historical estimates were also extended to the same incremental exercise performed in hot ambient temperatures. In short, it was indicated that the significant skin hyperaemia seen at rest would be progressively reduced during incremental exercise (Rowell, 1974). Contrary to this, we found that when incremental exercise was followed shortly after prolonged constant exercise (incremental exercise test 2), the concomitant 3‐fold forearm blood flow and skin hyperaemia at baseline was maintained at a similarly high level throughout the duration of incremental exercise. The extent of forearm hyperaemia seen during incremental exercise test 2 was greater than during the incremental exercise tests with a normal starting internal temperature (tests 1 and 3; 452 ± 113 vs. 285 ± 52 mL/min at 80% *W*_peak_), with this response being coupled to an elevation in core temperature (Figure 4). ${\dot{Q}}_{\mathrm{forearm}}$ and ${\dot{Q}}_{\mathrm{skin}}$ also increased and remained high during prolonged submaximal exercise, in the face of unchanged forearm muscle oxygen saturation (Figure 3g), similar to previous observations during prolonged, sub‐maximal exercise in normothermic and warm environments (Ooue et al., 2008; Padilla et al., 2011; Simmons et al., 2011). Collectively, the present data refute the longstanding view that profound reductions in non‐exercising limb tissue and skin perfusion occur during incremental exercise in the heat to volitional exhaustion.

The blood oxygen data shed further light on the stimuli triggering the increase in global forearm and skin hyperaemia. In the present investigation, venous blood responses reflect changes in the cutaneous circulation as samples were withdrawn from a catheter inserted into the median cubital vein in the anterograde direction. Two scenarios are plausible in the conditions of the present study: (1) an increased blood flow in both the skin and muscle of the non‐exercising forearm, and (2) increased skin blood flow, with either maintained or reduced forearm muscle blood flow. In the first scenario, an increased non‐exercising forearm muscle blood flow would generally be a response to increased metabolic demand, and thus a rise in forearm ${\dot{V}}_{O_{2}}$. This is, however, unlikely to be the case, as whole‐arm ${\dot{V}}_{O_{2}}$ has been shown to be maintained at baseline values during light to intense exercise, with only a small increase being observed at near‐maximal leg exercise (Calbet et al., 2007; González‐Alonso et al., 2015). The hand position in that multi‐study investigation was however maintained overhead, gripping the crank handles of a second arm cranking ergometer, and feasibly contracting to stabilize the torso when upright leg exercise was very intense. In contrast, in the present study, the arm was kept relaxed at the side of the participant throughout incremental and prolonged, constant semi‐recumbent cycling exercise (Figure 1). Our estimations of forearm aerobic metabolism using the measured forearm blood flow and a‐vO_2_ diff suggest that forearm ${\dot{V}}_{O_{2}}$ was stable among conditions of the present study (i.e. ∼7–8 mL/min). This notion is supported by the findings that forearm muscle oxygen saturation (Figure 3g) and perfusion in the brachial vein (deep vein) and forearm muscle do not change (Johnson & Rowell, 1975; Ooue et al., 2008), but blood flow in the brachial artery and superficial (basilic) vein (reflecting the skin circulation) increases substantially (Ooue et al., 2008) during prolonged leg exercise with concomitant hyperthermia.

**FIGURE 5:**
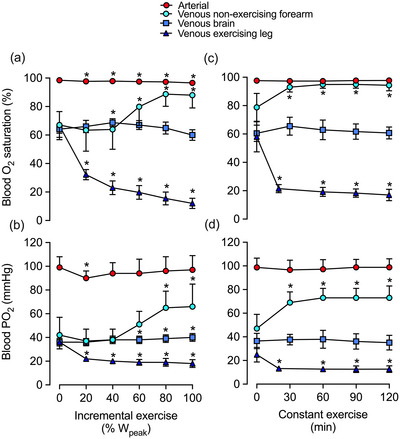
A comprehensive overview of blood oxygen saturation and the partial pressure of oxygen ($P_{O_{2}}$), in different vascular beds, collated from four studies, during incremental and constant, sub‐maximal exercise. Arterial, resting forearm, brain and exercising leg blood oxygen saturation during incremental (a, b) and constant, sub‐maximal (c, d) exercise in the heat. Arterial and resting forearm data are from study 1 as presented in the current paper. Brain O_2_ saturation and $P_{O_{2}}$ are redrawn from Trangmar et al. (2015, 2014). Exercising leg arterial and venous O_2_ saturation and $P_{O_{2}}$ are redrawn from González‐Alonso et al. (1998) and Trangmar et al. (2017). Arterial oxygen saturation decreases slightly from rest to peak exercise, whereas $P_{O_{2}}$ remains unchanged. Constant load submaximal exercise elicits little change in arterial oxygen saturation or $P_{O_{2}}$. Inactive‐forearm oxygen saturation and $P_{O_{2}}$, reflecting skin perfusion, are stable from rest to moderate intensity exercise, before increasing and stabilizing at a high level at near maximal exercise intensities. Comparatively, deep tissue (leg venous) oxygen saturation and $P_{O_{2}}$ decline substantially during both incremental and constant‐load exercise. In contrast, brain oxygen saturation increases slightly at moderate intensity exercise, before returning to baseline levels thereafter, whereas $P_{O_{2}}$ remains stable throughout incremental exercise, with no alterations in either variable during constant load exercise. Values are means ± SD for nine participants. *Differences from rest, *P* < 0.05.

The second scenario in which increases in skin circulation largely or entirely accounts for the observed forearm hyperaemia is therefore a more likely possibility. Several observations support this notion. First, we found that venous blood O_2_ content increased to a high level beyond submaximal exercise intensities, and during prolonged, constant exercise, before stabilizing (Figure 2a). Second, forearm skin a‐vO_2_ diff, while initially unchanged at lower exercise intensities, declined at volitional exhaustion to a level more than one‐third of the value seen in early exercise (66 ± 29 mL/L vs. 17 ± 13 mL/L). Third, the observed increase in blood O_2_ content and decrease in forearm skin a‐vO_2_ diff during incremental and prolonged exercise were seemingly proportional to the increase in skin and forearm blood flow (Figure 3a). Finally, forearm (antecubital) venous O_2_ saturation and $P_{O_{2}}$ increased in concert with the rise in forearm blood flow (Figure 5a, b). These findings collectively substantiate a close coupling between alterations in forearm perfusion and changes in cutaneous oxygenation.

### 4.2 Blood flow control in the non‐exercising limbs

It is well‐established that the peripheral haemodynamic adjustments occurring during incremental exercise and prolonged, constant exercise are closely coupled to the metabolic and thermoregulatory needs of tissue and organs with varied contributions of peripheral and central regulatory mechanisms (Laughlin et al., 2012; Rowell, 1974). To further support the idea that the local control of blood circulation differs among bodily tissues and organs, Figure 5 presents a comprehensive overview of arterial oxygen saturation and $P_{O_{2}}$ as well as the corresponding venous values from the non‐exercising forearm, exercising leg and brain during a range of exercise intensities and durations, obtained in the present and other comparable studies (González‐Alonso et al., 1998; Trangmar et al., 2015, 2014, 2017). Of note are the substantial differences in the venous oxygen saturation and $P_{O_{2}}$ responses among the non‐exercising forearm, the exercising leg tissues and the brain at high exercise intensities and when exercise is prolonged (Figure 5a, b). The non‐exercising forearm shows marked increases in oxygen saturation and $P_{O_{2}}$, the exercising skeletal muscles of the leg profound declines whereas the brain responses are modest or sustained. Taken together, these data indicate that perfusion in non‐exercising and exercising limbs increases during incremental and constant load exercise chiefly in response to thermoregulatory and metabolic stimuli, respectively, and suggest that, regardless of the primary stimuli, the mediating regulatory signals modulate the vasoconstrictor effects of markedly enhanced sympathoadrenal activity.

Peripheral and central thermal and non‐thermal mechanisms, working primarily via changes in circulatory vasoactive signals and sympathoadrenal activity, have been implicated in the regulation of local cutaneous vasomotor tone and blood flow (Blair et al., 1961; Charkoudian, 2010; Chiesa et al., 2015; Green, Cheetham, Mavaddat, et al., 2002; Johnson, 1986, 1998; Kalsi et al., 2017; Kellogg et al., 1991b, 1995; Laughlin et al., 2012; Ooue et al., 2008; Padilla et al., 2011; Rowell, 1990; Simmons et al., 2011). In reference to the role of local thermosensitive mechanisms, forearm skin temperature in the present study remained stable at ∼35°C, consistent with the dynamics of arm and forearm blood temperature observed during incremental and prolonged leg cycling exercise (González‐Alonso, Calbet, et al., 1999; González‐Alonso et al., 2015). This indicates that that local skin hyperthermia was not a primary stimulus for the rise in cutaneous perfusion. Instead, the herein observed positive relationship between the increase in forearm and skin blood flow and the rise in core temperature during the three incremental exercise bouts and the constant load exercise bout (Figure 4a–c) lends support to a centrally mediated vasodilatory response (Boulant, 2000; Charkoudian, 2003). It is well‐known that increasing internal body temperature induces sympathetically mediated reflex active vasodilatation, elevating skin vascular conductance and skin blood flow (Kellogg et al., 1995; Laughlin et al., 2012). A number of circulating vasodilator substances have been implicated in the reflex active vasodilatation of the cutaneous circulation with increasing internal temperature (Johnson et al., 2014), including nitric oxide (Green, Cheetham, Mavaddat, et al., 2002; McNamara et al., 2014; Padilla et al., 2011; Simmons et al., 2011), ATP (Fujii et al., 2015; Kalsi & González‐Alonso, 2012; Kalsi et al., 2017) and vasoactive intestinal peptide and histamine (Wong & Hollowed, 2017), but their interaction with circulating and neural vasoconstrictor signals is not well understood. There is evidence that the relationship between skin blood flow and core temperature is attenuated when internal temperature exceeds ∼38°C (Brengelmann et al., 1977; González‐Alonso, Teller, et al., 1999; Johnson, 1986; Johnson & Park, 1981; Kellogg et al., 1990; Nadel et al., 1979; Smolander et al., 1987) and that increases in circulating catecholamines during exercise via intravascular infusion can reduce cutaneous blood flow and cause an abrupt increase in core temperature (Mora‐Rodríguez et al., 1996). However, data from the present study suggest that vasodilator activity prevails in the cutaneous circulation to afford an increase and subsequent maintenance of high blood flow levels, in the presence of a heightened systemic sympathoadrenal drive. Although the precise signalling pathways underpinning this phenomenon warrant further investigation, it seems plausible that increases in vasoactive substances (such as ATP, which has vasodilator and sympatholytic properties) (Charkoudian, 2010; Duff et al., 1954; González‐Alonso et al., 2015; Kalsi et al., 2017; Rosenmeier et al., 2004; Trangmar et al., 2017, 2015) and metabolites (i.e., marked increases in forearm venous lactate concentration, accompanied by significant reductions in blood pH, $P_{CO_{2}}$, HCO_3_^−^ and acid–base excess; Tables 1, 2, 3) modulate the effects of vasoconstrictor signals in the vasculature of non‐exercising limb tissues, a phenomenon resembling the functional sympatholysis occurring in exercising muscle.

### 4.3 Methodological considerations and limitations

Owing to the time gap between studies, forearm blood flow data and blood parameters are from two cohorts of participants. However, we designed study 2 to completely match the protocols from study 1, such that we expect the same pattern of responses if data were collected in the same participants. We acknowledge the small sample size for both studies; an increased sample size may have identified other statistical differences currently not seen (e.g. the fall in ${\dot{Q}}_{\mathrm{forearm}}$ from rest to 20%). Based on the strength of the significance of the existing data, we do not feel this would affect the important conclusion on whether or not ${\dot{Q}}_{\mathrm{forearm}}$ and ${\dot{Q}}_{\mathrm{skin}}$ decline at near‐maximal exercise intensities. Due to the technical limitations of ultrasound, we could not obtain direct measures of forearm muscle blood flow during exercise, and so we cannot conclusively determine non‐exercising muscle blood flow during leg exercise. However, the complimentary measures of forearm a‐vO_2_ diff, skin blood flow and muscle oxygenation and the forearm arterial, venous (deep and superficial) and muscle blood flow from the literature (Johnson & Rowell, 1975; Ooue et al., 2008), discussed in‐depth above, provide sufficient support for our conclusions. We chose a combination of exercise and ambient temperature similar to previous work from our laboratory and aligned to a typically hot terrestrial temperature. Raising skin temperatures to much hotter levels, for instance by direct skin heating using water‐perfused garments, might yield different findings. However, unlike normal exercise in the heat, this approach also increases non‐exercising muscle temperature, tissue oxygenation and muscle blood flow (Heinonen et al., 2011; Kalsi et al., 2017; Koch Esteves et al., 2021; Pearson et al., 2011; Watanabe et al., 2024), leading to similar elevations in peak ${\dot{Q}}_{\mathrm{forearm}}$ (i.e., ∼500 mL/min) as seen here (Watanabe et al., 2024). The present findings that ${\dot{Q}}_{\mathrm{forearm}}$ and ${\dot{Q}}_{\mathrm{skin}}$ remained high and a‐vO_2_diff was very low throughout the maximal test 2 suggest that substantial declines in cutaneous blood flow are unlikely to happen when maximal exercise is initiated in a condition of significant hyperthermia and skin hyperperfusion. Finally, measurements of ${\dot{Q}}_{\mathrm{skin}}$ were made at only one site (i.e., the skin of the forearm) and only in male participants. Future studies could look to explore skin haemodynamics at other locations of the body in both sexes to identify if our findings in non‐exercising limbs are confirmed in other skin areas responsive to exercise and heat stress (e.g., the forehead) (Trangmar et al., 2015, 2014; Watanabe et al., 2024, 2020).

### 4.4 Conclusions

This study systematically investigated the non‐exercising forearm tissue and skin haemodynamic and oxygenation responses to a range of two‐leg exercise intensities and durations in the heat. Rather than declining, we found that skin blood flow increases to a high level, and is stable thereafter, during high‐intensity lower‐limb cycling exercise, accompanying proportional reductions in oxygen extraction from the cutaneous circulation. Mechanistically, the rise in forearm and skin blood flow with increasing internal body temperature was coupled to proportional changes in blood oxygen, reflecting a thermally mediated hyperaemia. The present findings challenge the widespread notion that skin blood flow declines markedly during dynamic exercise in the high‐to‐maximal intensity domains because of enhanced sympathoadrenal vasoconstrictor activity.

## AUTHOR CONTRIBUTIONS

This study was performed at Human, Environmental and Exercise Physiology Laboratory, Brunel University of London, Uxbridge, UK. Steven J. Trangmar and José González‐Alonso conceived and designed the research. Steven J. Trangmar and José González‐Alonso acquired the data. Steven J. Trangmar analysed the data. Steven J. Trangmar and José González‐Alonso interpreted the data. Both authors have read and approved the final version of this manuscript and agreed to be accountable for all aspects of the work in ensuring that questions related to the accuracy or integrity of any part of the work are appropriately investigated and resolved. All persons designated as authors qualify for authorship, and all those who qualify for authorship are listed.

## CONFLICT OF INTEREST

None declared.

## Data Availability

The data that support the findings of this study are available from the authors upon reasonable request.
